# Organization of Serotonergic Cell Populations in the Brain and Spinal Cord of the Short-Lived African Turquoise Killifish

**DOI:** 10.3390/biology14091206

**Published:** 2025-09-06

**Authors:** Laura González-Llera, Álvaro J. Arana, Laura Sánchez, Ramón Anadón, Antón Barreiro-Iglesias

**Affiliations:** 1 Departamento de Bioloxía Funcional, Facultade de Bioloxía, Universidade de Santiago de Compostela, 15782 Santiago de Compostela, Spain; laura.gonzalez.llera@rai.usc.es (L.G.-L.);; 2 Aquatic One Health Research Center (ARCUS), Universidade de Santiago de Compostela, 15782 Santiago de Compostela, Spain; 3 Department of Zoology, Genetics and Physical Anthropology, Faculty of Veterinary Science, Universidade de Santiago de Compostela, 27002 Lugo, Spain; alvaro.arana@usc.es (Á.J.A.); lauraelena.sanchez@usc.es (L.S.); 4 Preclinical Animal Models Group, Health Research Institute of Santiago de Compostela (IDIS), 15706 Santiago de Compostela, Spain

**Keywords:** killifish, serotonin, aging, neurodegeneration

## Abstract

The African turquoise killifish is a short-lived vertebrate that is gaining interest as a model for understanding degenerative processes associated with aging, including neurodegeneration. However, due to its relatively recent adoption in laboratory research, detailed studies on the adult neuroanatomy of the killifish, particularly regarding the organization of neurotransmitter systems and their age-related changes, remain limited. Here, we study the organization of serotonergic cell populations in the brain and spinal cord of juvenile/young adult (1–3-months-old) and geriatric (5–6-months-old) killifish using anti-serotonin immunofluorescence. Our findings reveal that the overall organization of serotonergic cell populations in killifish closely resembles that of most teleosts, including pineal, pretectal, hypothalamic, rapheal and spinal cord neuronal populations. Of note, adult killifish also present a population of serotonergic neurons in the dorsolateral isthmus, which has been only described in a few other fish species. Aged killifish showed a similar organization of serotonergic cell populations to that of juveniles/young adults, with the major difference being the loss of serotonin immunoreactivity in pretectal cells of 6-month-old fish. Our study provides a neuroanatomical framework for future investigations into the serotonergic system in this emerging vertebrate model of aging.

## Simple Summary

The African turquoise killifish, which is the shortest-lived vertebrate species bred in captivity, is emerging as a valuable vertebrate model for studying the effects of aging on the nervous system. Here, we studied the organization of serotonergic neuronal populations in the brain and spinal cord of juveniles and young adult killifish, and the changes that occur in geriatric animals. The killifish shows a similar organization of the serotonergic system as compared to other teleosts, but of note is the presence of an unusual serotonergic cell population in the dorsolateral isthmus. Our findings show a decline in serotonin expression in cells of the pretectal region in aged animals and provide a neuroanatomical foundation for future research on the serotonergic system in the killifish model.

## 1. Introduction

The brains of vertebrates exhibit both a conserved basic pattern of organization in its main regions resulting from the common plan of neurogenetic developmental programs, together with large variations affecting size and specialization in different regions reflecting a complex evolution of groups and species. Teleosts comprise the largest number of species within vertebrates (more than 30,000), having diversified since the early Mesozoic era [1,2]. Here, we study the brain serotonergic system of a small, short-lived teleost fish that in recent years has been established as a model for aging studies (reviewed in [3,4,5]). The African turquoise killifish (*Nothobranchius furzeri*) is a species belonging to the order Cyprinodontiformes, pertaining to the superorder of advanced teleosts known as Acanthopterygian. This short-lived species inhabits ephemeral ponds in south-east Africa and lays protected eggs with embryos that may survive the dry season until the next rainy season [6]. Then, the embryo resumes development and, after hatching, gives rise to an alevin (juvenile) that grows into a sexually mature adult and in a few months (typically 5 to 7 months depending on the strain) suffers fast aging and dies. Ease of management, small size and short life make the African turquoise killifish a highly valuable vertebrate model for aging studies.

Discovered as a serum vasoconstrictor factor [7], serotonin (5-hydroxytryptamine) is an indoleamine mainly produced by enterochromaffin cells of the gut and some brain neurons that use it as a neurotransmitter. Its presence in the brain is known from biochemical and histofluorescent studies since the 1950s, and its distribution in the central nervous system (CNS) has been reported in a number of vertebrate and invertebrate species using immunohistochemical methods with antibodies raised against serotonin–protein conjugates [8]. In fishes, there are immunohistochemical studies revealing the distributions of serotonergic neurons in lampreys [9,10,11,12,13,14], elasmobranchs [15,16,17,18,19], and actinopterygians (cladistians [20]; sturgeons [21]; holosteans [22]; teleosts [23,24,25,26,27,28,29,30,31,32,33,34] and lungfishes [35]). In fish species, the serotonergic raphe nuclei and hypothalamic populations are the most conspicuous (see [8]). Serotonin released by serotonergic neurons at synapses binds to serotonin receptors, although serotonin also acts extra-synaptically [36,37]. As a promiscuous neurotransmitter, serotonin is involved in pleiotropic brain functions, via different families of serotonin receptors (presynaptic and postsynaptic) [38,39]. Numerous behaviours and functions appear to be modulated by serotonin during development and adulthood, including, among others, locomotion, aggression, fear, anxiety or neuronal regeneration [8,31,40,41,42,43].

In the aging brain of mammals, the serotonergic system experiences changes that affect levels of serotonin as well as levels of serotonin receptors, serotonin transporters and tryptophan hydroxylase, the rate-limiting serotonin-synthesizing enzyme (reviewed in [44]). In general, levels of serotonin in rodent brains are reduced with age in some brain regions, and levels of receptors are also altered with age [44]. Morphological changes in aged serotonergic fibres have been also described in some brain areas in rodents [45,46,47,48]. Neuroanatomical studies of the serotonergic system during aging in other vertebrate groups are scant. An immunohistochemical study performed in a teleost reported some changes with aging in the preoptic serotonergic system of platyfish [49]. Recent biochemical studies in the brain of killifish reveal age-related changes in the level and metabolism of serotonin, as well as in expression levels of serotonin-related genes, and sex differences during aging [50,51]. These recent studies suggested that killifish is a suitable model for studying the aging-related changes in the serotonergic system and the role of serotonin in age-related behavioural dysfunctions. However, although the distribution of serotonergic neurons has been reported in several other teleost models (see above), there is no available description in the CNS of this emerging model for aging research, and only a recent study has revealed the presence of serotonergic cells in the killifish intestine [52]. Here, we present the first neuroanatomical description of the serotonergic neuronal populations in the brain and spinal cord of young and aged African turquoise killifish adults. Interestingly, our results only reveal very minor differences in the anatomical distribution of serotonergic neurons between young (2- to 3-month-old) and aged (5- to 6-month-old) adults. Our work provides an anatomical reference for future work on the serotonergic system in this short-lived vertebrate.

## 2. Materials and Methods

### 2.1. Animals and Immunofluorescence

Fourteen juvenile/adult killifish (*Nothobranchius furzeri*, GRZ strain) of different ages were used in this study: one 1-month juvenile (alevin), six 2–3-months old adults (three males and three females), and seven 5–6-month old adults (three males and four females). All animals belonged to the GRZ strain, originally collected in 1968 in Zimbabwe and maintained by inbreeding. Under standard laboratory conditions, this strain exhibits a median lifespan of approximately 4–6 months and a maximum lifespan of 7–8 months (reviewed in [53]). Animals were kept in the animal facility of the Faculty of Veterinary Medicine (University of Santiago de Compostela, Lugo, Spain; authorized with the REGA code ES270280346401) in a recirculating aquatic system that maintains the appropriate environmental conditions for this species, including controlled temperature, pH, salinity, and photoperiod. All procedures were carried out with the agreement of the bioethical committee of the University of Santiago de Compostela and the Xunta de Galicia. Fish were euthanized with 0.5% tricaine methanesulfonate (i.e., 0.5 g in 100 mL of fish water; MS-222; Sigma, Darmstadt, Germany) and through subsequent decapitation. The heads were fixed through immersion in 4% paraformaldehyde in 0.2 M phosphate buffer (pH 7.4) at 4 °C for 24–48 h. Then, brains and rostral spinal cords were dissected out, cryoprotected with a solution of 30% sucrose in phosphate-buffered saline (PBS), embedded in Neg-50™ (Thermo Scientific, Waltham, MA, USA), and frozen in liquid-nitrogen-cooled isopentane, and sections were obtained on an 18 μm thick cryostat in transverse (*n* = 12) or sagittal (*n* = 2) planes. Sections were collected on Superfrost plus glass slides (Epredia, Kalamazoo, MI, USA) and air-dried overnight. Then, sections were incubated for 24 h at room temperature with a rabbit polyclonal anti-serotonin antiserum (Immunostar, Hudson, WI, USA, Cat#: 20080; RRID:AB_572263; dilution 1:2500) in PBS. The slides were rinsed three times with PBS and incubated for 1 h with a Cy3-conjugated goat anti-rabbit IgG antibody (Jackson ImmunoResearch, West Grove, PA, USA, Cat#: 111-165-144; RRID:AB_2338006; dilution 1:500), and they were then rinsed in PBS and distilled water and mounted with Mowiol (Sigma, Darmstadt, Germany). All primary and secondary antibodies were diluted in PBS containing 15% normal goat serum and 0.2% Triton X-100.

### 2.2. Specificity of the Primary Antibody

The polyclonal anti-serotonin antiserum was raised against a serotonin–creatinine sulphate–BSA conjugate and has been used to reveal serotonergic structures in many vertebrates and invertebrates, including various species of lampreys, elasmobranchs and bony fishes [10,13,19,21,22,35,54,55,56,57]. Staining is not observed when performing a pretreatment of the diluted antibody with 25 μg of serotonin–BSA. Cross-reactivity studies indicate that the serotonin antiserum does not react with 5-hydroxytryptophan, 5-hydroxyindole-3-acetic acid and dopamine (Immunostar technical information). Controls omitting the primary antibody led to no staining at all. The staining pattern obtained in the killifish was consistent with that obtained in studies of other teleosts.

### 2.3. Imaging

Images of fluorescent labelled sections were taken with a Leica Stellaris 8 confocal microscope using the green excitation laser and selecting the appropriate emission and acquisition wave band intervals for the Cy3 fluorophore. Confocal optical stacks were taken at steps of 2.5 μm (10× and 20× dry objectives, N.A. 0.85) or 0.7 μm (40× oil immersion objective, N.A. 1.3) along the *z*-axis. Lightning adaptive deconvolution was used to improve resolution in some images taken with the 40× objective. Z-stack images were processed and studied with LAS X software (version 5.3.1; Leica, Wetzlar, Germany) or FIJI free software (version 2.16.0). Figure plates were composed using Adobe Photoshop 2025 (version 26.10; Adobe).

## 3. Results

**Figure 1:**
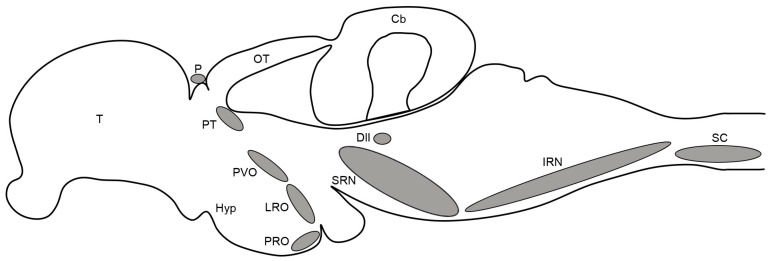
Schematic drawing of a lateral view of the adult killifish brain and rostral spinal cord showing an overview of the organization of serotonergic cell populations. Modified from [58]. For abbreviations, see list.

Given the absence of substantial differences in the distribution of serotonergic neurons in the brain and spinal cord between younger (1–3-month-old) and older (5–6-month-old) specimens, we provide a comprehensive characterization of the organization of serotonin-immunoreactive (5-HT-ir) neuronal populations in juveniles/young adults, highlighting only the minor changes that emerge with age. The following description of serotonergic cell populations will follow a rostral–caudal order in topological terms, i.e., secondary prosencephalon, diencephalon, rhombencephalon and spinal cord. A summary diagram illustrating the location of 5-HT-ir neuronal populations is provided in Figure 1.

**Figure 2:**
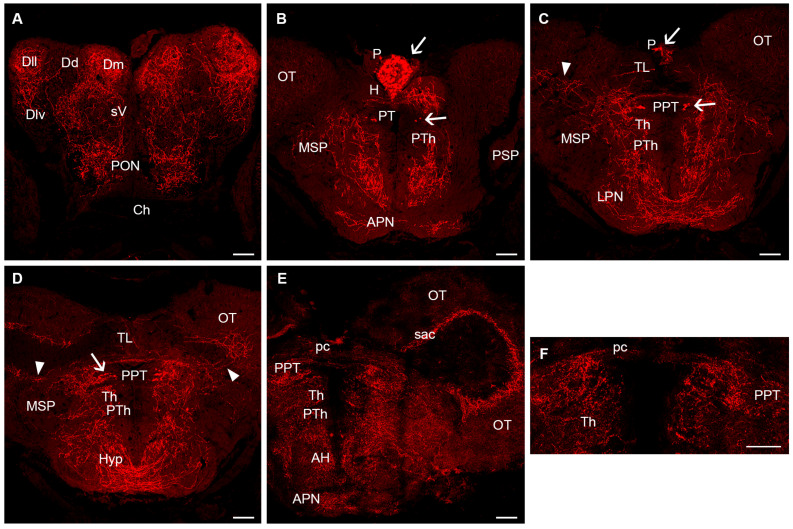
Serotonin immunoreactivity in the telencephalon, hypothalamus, diencephalon and mesencephalon of juvenile and adult killifish. (**A**). Photomicrograph showing the presence of extensive serotonergic innervation in different telencephalic areas in a juvenile killifish. (**B**,**C**). Photomicrographs showing the presence of 5-HT-ir cells in the pineal and pretectum of the juvenile killifish. (**D**). Photomicrograph showing the presence of 5-HT-ir cells in the pretectum (arrow), the presence of 5-HT-ir fibres crossing in the posterior commissure and of 5-HT-ir fibres entering the optic tectum (arrowheads) of the juvenile killifish. (**E**,**F**). Photomicrographs of a 6-month-old adult revealing the lack of 5-HT-ir cells in the pretectal region and presence of 5-HT-ir innervation in the optic tectum. Arrows indicate 5-HT-ir cells. Arrowheads indicate 5-HT-ir fibres entering the optic tectum from the pretectum. Dorsal is the top in all figures. Scale bars: 60 µm. For abbreviations, see list.

**Figure 3:**
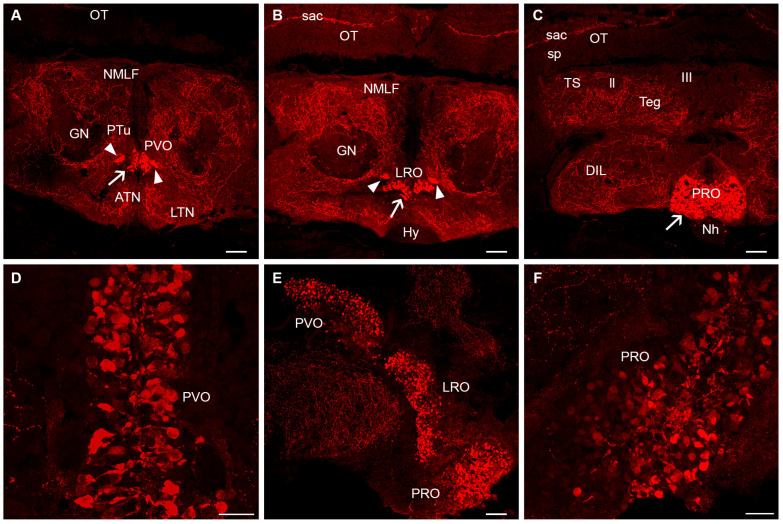
Serotonin immunoreactivity in the hypothalamus, diencephalon and mesencephalon of juvenile and adult killifish. (**A**). Photomicrograph showing the presence of 5-HT-ir CSF-c cells in the paraventricular organ of a juvenile killifish. (**B**). Photomicrograph showing the presence of 5-HT-ir CSF-c cells in the lateral recess organ of the juvenile killifish. (**C**). Photomicrograph showing the presence of 5-HT-ir CSF-c cells in the lateral recess organ of the posterior recess organ of the juvenile killifish. (**D**). Detail of the CSF-c cells of the paraventricular organ (40× objective and lightning adaptive deconvolution) in the 2-month-old adult killifish. (**E**) Photomicrograph of a sagittal section showing the presence of 5-HT-ir cells of the paraventricular, lateral recess and posterior recess organs of the 2-month-old adult killifish. (**F**). Detail of the CSF-c 5-HT-ir cells of the posterior recess organ in a sagittal section of the 2-month-old adult killifish. Arrowheads indicate the conspicuous 5-HT-ir tract common to the three circumventricular organs. Arrows indicate 5-HT-ir cells. Dorsal is at the top in all figures. Scale bars: (**A**–**C**,**E**): 60 µm; (**D**,**F**): 20 µm. For abbreviations, see list.

The secondary prosencephalon lacks 5-HT-ir cells in the olfactory bulb, telencephalon, optic-related region and rostral hypothalamus (Figure 2). These regions receive abundant serotonergic innervation, especially in the medial region of the olfactory bulbs containing the granule cells, the pallium, the subpallium, the central zone of the neuropil of the preoptic region and the rostral hypothalamus (Figure 2A–E). However, a number of 5-HT-ir cells of cerebrospinal fluid-contacting (CSF-c) type were observed in the caudal (dorsal) hypothalamus in the walls of the third ventricle and lateral and posterior hypothalamic recesses (Figure 3). These cell populations correspond to the paraventricular organ (rostral), periventricular nucleus of the lateral hypothalamic recess and periventricular nucleus of the posterior hypothalamic recess (Figure 3). These CSF-c 5-HT-ir cells show a ventricular dendrite ending as a small club in these nuclei (Figure 3D) except in the posterior recess, where intraventricular endings appear more complex (Figure 3F). In young adults, the CSF-c cell band of the paraventricular organ is continuous with that of CSF-c cells in the periventricular region of the lateral recesses. In 5- to 6-month-old adults, the population of the posterior recess organ becomes separated from that of the lateral recess, and the number of 5-HT-ir cells in these ventricular organs is clearly higher than in juveniles. A conspicuous 5-HT-ir tract of thin fibres common to the three circumventricular organs can be observed above the lateral recess organ and extending to a wide neuropil over the lateral region of the posterior recess organ (Figure 3A,B). Other targets of these circumventricular organs of the caudal hypothalamus were not identified.

In the diencephalon, 5-HT-ir neurons were observed in the pineal organ and pretectum (Figure 2B–D). The pineal organ contains numerous 5-HT-ir cells in the parenchyma, some showing short basal processes (Figure 2B,C). These cells were tentatively identified as pineal photoreceptors that do not appear to give rise to pinealofugal projections, i.e., they appear to be intrapineal neurons. A small group of 5-HT-ir neurons (1–6 cells per side in a few sections) was observed in the pretectum at the level of the posterior commissure (Figure 2B,C). These cells are contiguous to the fasciculus retroflexus and form part of the periventricular pretectum, which is located in the periventricular region near the lateral neuropil. Interestingly, in the 6-month-old fish (both males and females), the periventricular pretectum showed no 5-HT-ir cells, or they were very faintly stained (Figure 2E,F). The medial region of the pretectum contains numerous 5-HT-ir fibres associated with this 5-HT-ir periventricular population, and some fibres cross in the posterior commissure (Figure 2C–F). 5-HT-ir fibres from this region also extend toward the optic tectum or ventrally (Figure 2C–E).

The mesencephalon did not show any 5-HT-ir cell populations. Of note are the two large optic lobes of the killifish midbrain, in which the optic tectum receives numerous 5-HT-ir fibres, at least in part from the pretectal region (see above), that form a layer in the border between the periventricular and the central white regions, from which some thinner branched fibres ascend through the cell and fibre layers forming two or three loose tangential plexuses (Figure 2C–E and Figure 3B,C). We did not detect any obvious changes in the innervation of the optic tectum in the oldest adults, even despite the loss of 5-HT immunoreactivity in cells of the pretectal region (Figure 2E).

**Figure 4:**
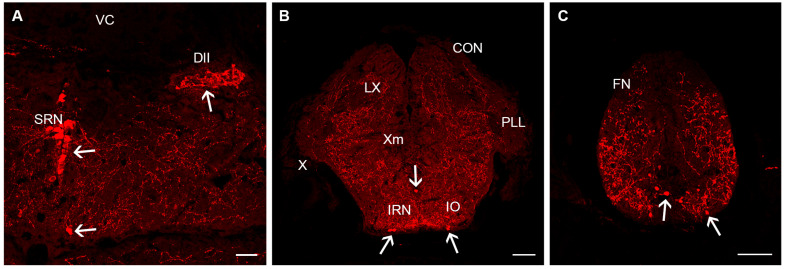
Serotonin immunoreactivity in the rhombencephalon and spinal cord of juvenile killifish. (**A**). Photomicrograph showing the presence of 5-HT-ir cells in the superior raphe nucleus and dorsolateral isthmus. (**B**). Photomicrograph showing the presence of 5-HT-ir cells in the inferior raphe nucleus. (**C**). Photomicrograph showing the presence of 5-HT-ir cells in the spinal cord. Arrows indicate 5-HT-ir cells. Dorsal is at the top in all figures. Scale bars: 60 µm. For abbreviations, see list.

The cerebellum consists of the cerebellar body flanked by the granular eminences and extending in a rostral portion inside the midbrain ventricle, the cerebellar valvula, and caudally in a poorly differentiated caudal lobe. The cerebellum lacks 5-HT-ir cells, but some 5-HT-ir fibres can be observed entering the granular eminences and cerebellar body, branching widely in the granular layer but showing a low density. 5-HT-ir fibres are scant in the valvula (Figure 4A) and caudal lobus.

Just behind the mesencephalo/rhombencephalic boundary (passing between the III and IV nerve motor nuclei and rostral to the interpeduncular nucleus), we observed a conspicuous population of 5-HT-ir cells in the median raphe corresponding to the superior raphe nucleus (Figure 4A). These neurons are located in the midline of the basal region, mostly in its upper half, and with a few neurons located more laterally or ventrally. This population extends caudally to the level of entrance of the trigeminal nerve and motor nucleus. Caudally to the facial nerve entrance, the number of 5-HT-ir neurons in the raphe are scant, but a few cells (typically one or two per hemisection) appear in the ventralmost (subpial) location laterally to the midline and occasionally in the ventral raphe. This scattered population corresponds to the inferior raphe nucleus and extends to the obex (Figure 4B).

In addition to these rapheal nuclei, a conspicuous population of small 5-HT-ir cells was observed in the isthmus, forming a lateral (horizontal) band of cells with irregular profiles (Figure 4A). The rostro-caudal extension of this dorsolateral isthmic nucleus is very short, only one to three transverse sections.

The spinal cord contains a sparse population of small 5-HT-ir cells scattered along the spinal cord near the medial longitudinal fascicle or ventral to it (Figure 4C).

## 4. Discussion

### 4.1. Comparative Neuroanatomy of the Serotonergic System in Fishes

The distribution of 5-HT-ir perikarya in the brain of fishes has been the subject of numerous studies (reviewed in [8]). Here, we discuss the distribution of serotonergic populations in killifish compared with other fishes in relation to the phylogeny. *Nothobranchius* belongs to Ovalentaria (a group of evolved teleosts that includes species of *Xiphophorus*, *Gambusia*, tilapia, *Oryzias*, etc.), which have been the subject of serotonin immunohistochemical studies.

The prosencephalon of adult *Nothobranchius furzeri* lacks serotonergic cells in the olfactory bulb, telencephalon, optic recess region and rostral hypothalamus. In this regard, present findings in the African turquoise killifish are similar to those reported in many fishes, including lampreys [9,10,11,13,59], elasmobranchs [15,17,19,60], cladistians [20], chondrosteans [21], holosteans [22], most teleosts (*Carassius auratus* [23]; *Salmo gairdneri* (=*Oncorhynchus mykiss*) [24]; *Gasterosteus aculeatus* [25]; *Xiphophorus* sp. [49]; *Gnathonemus petersii* [27]; *Apteronotus leptorhynchus* [28]; *Eigenmannia lineata* [61]; *Clarias gariepinus* [62]; *Dicentrarchus labrax* [29]; *Solea senegalensis* [30]; *Allenbatrachus grunniens*, *Ariopsis seemanni* and *Synodontis nigriventris* [57]) and lungfishes [35]. In the olfactory bulb, aggregations of serotonergic cells were reported in *Micropogonias undulatus*, *Gambusia affinis* and tilapia [33,34,63], some of them pertaining to Ovalentaria, which was not observed here in the adult killifish. A few serotonergic cells were observed in the telencephalon of a guitarfish [18] and *Gambusia affinis* [33]. The scant presence of telencephalic serotonergic populations suggests that they represent derived characters that appeared independently in a few fish lineages. In the optic recess region, serotonergic cells were reported in the preoptic nucleus of tilapia [32,34], as well as in cladistians [20], sturgeons [21] and holosteans [22], but not in most teleosts, including the killifish. The functional roles of these differences among species are, at present, unknown.

A number of 5-HT-ir cells of CSF-c type were observed in the caudal (dorsal) hypothalamus of killifish comprising the paraventricular organ (rostral), periventricular nucleus of the lateral hypothalamic recesses and periventricular nucleus of the posterior hypothalamic recesses, which show a similar organization in young and aged killifish. As noted, the CSF-c cell serotonergic population of the paraventricular organ is continuous with that of the lateral recesses, but the population of the posterior recess was somewhat separated from that of the lateral recess. These periventricular populations show more abundant CSF-c cells in aged adults than in juveniles, which could be related to the presence of an adult neurogenic niche (EdU+) reported in the ventricular system of the adult hypothalamus [64]. A similar organization of this serotonergic circumventricular complex has been described in all studied teleost species [8], and they are similar to those described in other non-teleost bony fishes (chondrosteans [21]; holosteans [22]) and in elasmobranchs [19,54]. In lampreys, the distinction of subpopulations of serotonergic hypothalamic cells is unclear: a long, continuous band of CSF-c serotonergic cells extends throughout the caudal hypothalamus to the so-called mammillary recess [10]. In lungfishes, too, a long periventricular organ without segregated parts is found along the hypothalamus [35]. The reasons for these anatomical differences between fish groups are not well understood. Whilst serotonergic hypothalamic populations have been mainly studied using anti-serotonin immunohistochemistry, in a few fish species they were also studied with tryptophan hydroxylase (*tph*) in situ hybridization, showing that hypothalamic serotonergic cells have this key serotonin-synthesizing enzyme (sea lamprey *tph* [59]; zebrafish *tphD* [65]; rainbow trout *tph1* [66]). The 5-HT-ir tract of thin fibres common to these circumventricular organs seen above the lateral recess organ and extending to a wide neuropil over the lateral region of the posterior recess organ appears to also be a conserved feature in teleosts.

In the goldfish, Atlantic croaker, Senegalese sole, tilapia, Gambusia and midshipman, 5-HT-ir cells have been reported in the adenohypophysis [23,30,31,32,33,49,61,63], which was not noted in the pituitary of killifish. In elasmobranchs, 5-HT-ir cells were also noted in the adenohypophysis [15,17]. Actual roles of serotonin in these hypophysial serotonergic cells are not known, but it may act as a neurotransmitter or hormone [15].

Numerous 5-HT-ir cells have been reported in the habenula of *Porichthys notatus* (midshipman; [32]), which has not been observed in most teleosts. Only Ekström and Ebbesson [26] reported serotonergic neurons in the left habenula of sockeye salmon fry (*Oncorhynchus nerka*), a population that appears to be transient. Similar habenular populations were not found in other teleosts, including here in the killifish, although the transient presence of serotonergic cells has been reported in the habenula of an elasmobranch [19]. Whereas no serotonergic population was observed in killifish habenula, conspicuous asymmetrical serotonergic innervation of a region of the habenula (more extensive in left habenula) was noted (Figure 2B). In zebrafish, the central part of the habenula is targeted by serotonergic fibres arising from the raphe nuclei [31], and this may also occur in killifish. The left habenula receives innervation from the parapineal organ in teleosts and lampreys [67,68], but these fibres are probably not serotonergic.

5-HT-ir cells were also observed in the killifish pineal organ. By their morphology, these cells appear to represent pineal photoreceptors without pinealofugal projections that accumulate serotonin, similar to those reported in the pineal of other teleosts [26,69], cladistians [20], holosteans [22] and lampreys [10,70,71]. In zebrafish and other teleosts, two types of pineal photoreceptors are distinguishable by the type of parapinopsin (parapinopsin 1 or 2) present [72]. In photoreceptor cells expressing parapinopsin 2, serotonin immunoreactivity and expression of *tph* and *aanat* are also colocalized, which is interpreted as these cells being melatonin-expressing photoreceptors. They synthesize and accumulate serotonin as a precursor for the synthesis of melatonin by the AANAT [72]. In pike and zebrafish, the synthesis of melatonin by the pineal is regulated by a circadian clock [73]. In the turquoise killifish, the expression of circadian clock gene *bmal1* and the clock system appears largely confined to the pineal organ [74].

In the African turquoise killifish, a group of 5-HT-ir neurons was observed in the pretectum of young adults below and lateral to the posterior commissure and subcommissural organ. These cells are adjacent to the fasciculus retroflexus and form part of the periventricular pretectum. A similar serotonergic nucleus has been reported in most teleosts (*S. gairdneri* [24,27]; *G. petersii* [75]; *O. nerka* [26]; *A. burtoni* [32,56], *G. affinis* [33]; *A. grunniens*, *A. seemanni* and *S. nigriventris* [57]). Similar serotonergic pretectal/thalamic populations have also been reported in lampreys [9,10,55], elasmobranchs [19,60], chondrosteans [21], holosteans [22] and lungfishes [35], indicating a high degree of evolutionary conservation.

The optic tectum lacks serotonergic cell populations in the African turquoise killifish, as reported in most teleost fishes (*Carassius auratus* [23]; *Salmo gairdneri* [24]; *Gasterosteus aculeatus* [25]; *Xiphophorus* sp. [49]; *Gnathonemus petersii* [27]; *Apteronotus leptorhynchus* [28]; *Eigenmannia lineata* [61]; *Clarias gariepinus* [62]; *Dicentrarchus labrax* [29]; *Solea senegalensis* [30]; *Allenbatrachus grunniens*, *Ariopsis seemanni* and *Synodontis nigriventris* [57]). To our knowledge, the presence of serotonergic neurons in the optic tectum was only reported in a mosquitofish (*Gambusia affinis* [33]), in a lamprey (*Lampetra fluviatilis* [9]), and in lungfishes (*Protopterus dolloi* and *Neoceratodus forsteri* [35]). Whereas the presence of serotonergic cells in the tectum probably represents an innovation of a few species, in all fishes, the optic tectum receives abundant serotonergic fibres, although layering patterns of innervation vary among species. The origin of the serotonergic fibres innervating the tectum was not traced in most fish species. Studies in zebrafish using transgenic lines expressing a green fluorescent protein in serotonergic raphe neurons revealed that the optic tectum is not innervated by serotonergic raphe cells [8,31]. On the other hand, cells of the periventricular pretectum project to the optic tectum in tilapia [76] and zebrafish [77]; thus, a possible origin of tectal fibres in these teleosts is the serotonergic pretectal population. Since the serotonergic system appears rather well conserved in teleosts [8], it is probable that these data in tilapia and zebrafish also apply to the killifish, but this needs to be assessed experimentally in the future using neuronal tracers.

In the killifish rhombencephalon, the most conspicuous serotonergic cells are raphe cells located dorsal to and behind the interpeduncular nucleus, with a few serotonergic neurons located more laterally or ventrally, and until the level of the trigeminal motor nucleus. These populations correspond to the superior and medial raphe nuclei. Caudally to the facial nerve entrance, scarce 5-HT-ir cells appear in subpial location laterally to the midline and occasionally in the ventral raphe, corresponding to ill-defined intermediate/inferior raphe nuclei that extend to the spinal cord. This pattern of serotonergic raphe populations is like that reported in other teleosts [24,25,27,28,29,32,33,62]. Studies with transgenic zebrafish reveal that axons of serotonergic raphe cells project to most brain regions and the spinal cord, with some exceptions, such as the optic tectum as indicated above [8,31], and it is probable that the same occurs in killifish, although future hodological studies should confirm this.

In addition to these raphe nuclei, a population of 5-HT-ir cells was observed in the dorsolateral isthmus of killifish, forming a conspicuous lateral band of cells. Whether this population is mesencephalic or rhombencephalic was not assessed, but it probably corresponds to serotonergic nuclei described in a similar location (dorsolateral isthmus) in salmonids [24,26], *Gasterosteus aculeatus* [25] and *D. labrax* [29]. In sturgeons, a similar region is richly innervated by 5-HT-ir fibres but without immunoreactive neurons [21]. In the future, it would be of great interest to analyse the role of this serotonergic cell population in the brain of these specific teleost species, like the killifish.

The spinal cord of killifish contains sparse populations of small 5-HT-ir cells scattered near the medial longitudinal fascicle or ventral to it. The presence of serotonergic spinal cells has been reported in a variety of fish species including lampreys [10,59,78], elasmobranchs [15,16,19,79], cladistians [20], sturgeons [21], holosteans [22], teleosts [80], and lungfish [35]. Its role as a modulator of the central pattern generator for locomotion has been well studied in lampreys [78] and zebrafish [81].

### 4.2. Some Considerations on the Serotonergic System in Aging Killifish

Previous work in the aging killifish revealed decreased serotonin levels in whole brain extracts between 2- and 7-month-old killifish of the ZMZ1001 strain using HPLC methods [51]. Our neuroanatomical data show a loss of serotonin immunoreactivity in pretectal cells of 6-month-old killifish of the GRZ strain, which could reveal one of the reasons for the decrease in serotonin levels reported by Evsiukova et al. [51]. Whether there is a decrease in serotonin levels in other neuronal populations or fibre systems was not determined with the immunofluorescence method used in our study. Future studies using more quantitative approaches could assess this.

Most studies of the serotonergic system in fishes are concerned with the distribution of serotonin in adult animals, whereas studies of the development of the serotonergic system are centred on a few species: the lamprey *Petromyzon marinus* [10,11], the catshark *Scyliorhinus canicula* [19], the three-spined stickleback *Gasterosteus aculeatus* [82], the brook trout *Salvelinus fontinalis* [83] and the zebrafish *Danio rerio* [8,31,84,85,86]. To our knowledge, studies of neuroanatomical changes in the serotonergic system in aging fish are limited to the brain and pituitary in platyfish (*Xiphophorus*) [49]. These authors found serotonin expression in cells of the preoptic nucleus from 18-month-old platyfish onwards as the main change in the brain related to senescence, as well as increasing the expression of serotonin in pituitary gonadotropes. In the African turquoise killifish, however, no expression related to aging was observed in cells of the preoptic region, and in this respect, both species differ clearly. These authors proposed that the changes observed in aging platyfish were related to reproductive senescence. Another difference is the apparent fading of serotonin immunoreactivity in the pretectal nucleus perikarya of aging killifish, not noted in aging platyfish [49]. Since no clear changes were observed in the distribution of serotonergic fibres in the killifish optic tectum (presumably originating from pretectal cells; see above), the loss of immunoreactivity in pretectal perikarya might be related to changes in axonal transport and/or synthesis of serotonin in perikarya associated with aging. Despite both species appearing to be closely related in phylogeny (platyfish and killifish are both Cyprinodontiformes), they do not share the same changes in serotonin expression observed with aging, which suggests that findings in a fish species may not be applicable to other species and comparisons need be carried out with caution. Studies of degeneration in other CNS systems of the aging killifish suggest the maintenance of the core circadian clock system in the pineal organ [74], a loss of catecholaminergic cells in the locus coeruleus [87] and posterior tuberculum [87,88,89] or the upregulation of GFAP in brain radial glia [64].

## 5. Conclusions

Previous biochemical/transcriptomic studies revealed changes in serotonin levels, in key enzyme activity and in the expression of serotonergic genes in the aging killifish CNS [50,51], suggesting it could be a vertebrate model of interest for understanding the effects of aging in the serotonergic system or the role of serotonin in neurodegenerative processes and in behavioural changes related to aging. Our study provides the first anatomical description of the organization of serotonergic cell populations in the adult killifish brain. Our results reveal a similar organization of these cell populations as compared to most teleosts, including the conspicuous hypothalamic and rapheal neuronal populations. Of note, juvenile/adult killifish present a population of serotonergic cells in the dorsolateral isthmus, which has been only described in a few species. Aged killifish showed a similar organization of serotonergic cell populations to that of juveniles/young adults, with the major difference being the loss of serotonin immunoreactivity in pretectal cells of 6-month-old fish. Whether this feature contributes to the decrease in visual acuity previously reported in the aging killifish [90] should be assessed in future studies. We believe that our study provides an anatomical framework for further work using the killifish as a model for studying the consequences of aging on the serotonergic system.

## Data Availability

Raw imaging data are available from the authors upon reasonable request.

## Abbreviations

The following abbreviations are used in the figures:

AH	anterior hypothalamus
APN	anterior preglomerular nucleus
ATN	anterior tuberal nucleus
Cb	Cerebellum
Ch	optic chiasm
CON	caudal octavolateralis nucleus
D	dorsal telencephalic area (pallium)
Dd	dorsal zone of D
DIL	diffuse nucleus of the inferior lobes
DlI	dorsolateral isthmus
Dll	latero-lateral zone of dorsal telencephalon
Dlv	ventro-lateral zone of dorsal telencephalon
Dm	medial zone of dorsal telencephalon
FN	funicular nucleus
GN	glomerular nucleus
H	habenula
Hy	hypophysis
Hyp	hypothalamus
IO	inferior olive
IRN	inferior raphe nucleus
III	oculomotor nucleus
ll	lateral leminiscus
LPN	lateral preglomerular nucleus
LRO	lateral recess organ
LTN	lateral tuberal nucleus
LX	vagal lobe
MSP	magnocellular superficial pretectal nucleus
Nh	neurohypophysis
NMLF	nucleus of the medial longitudinal fascicle
OT	optic tectum
P	pineal organ (epiphysis)
pc	posterior commissure
PLL	posterior lateral line nerve
PON	preoptic nucleus
PRO	posterior recess organ
PSP	parvocellular superficial pretectal nucleus
PT	pretectum
PPT	periventricular pretectum
PTh	prethalamus (ventral thalamus)
PTu	posterior tubercle
PVO	paraventricular organ
sac	stratum album centrale (of the OT)
SC	spinal cord
sp	stratum periventriculare (of the OT)
SRN	superior raphe nucleus
T	telencephalon
Teg	tegmentum
Th	thalamus (dorsal thalamus)
TL	torus longitudinalis
TS	torus semicircularis
V	ventral telencephalic area (subpallium)
VC	valvula cerebelli
sV	subcommissural zone of V
X	vagal nerve
Xm	vagal motor nucleus
